# Development and validation of an interpretive guide for PROMIS scores

**DOI:** 10.1186/s41687-020-0181-7

**Published:** 2020-02-28

**Authors:** Nan E. Rothrock, Dagmar Amtmann, Karon F. Cook

**Affiliations:** 1 Department of Medical Social Sciences, Feinberg School of Medicine of Northwestern University, 625 N. Michigan Ave Suite 2700, Chicago, IL 60660 USA; 2 Department of Rehabilitation Medicine, University of Washington, Seattle, WA USA

**Keywords:** Patient-reported outcomes, PROMIS, Item response theory

## Abstract

**Background:**

Accurate score interpretation is required for the appropriate use of patient-reported outcome measures in clinical practice.

**Objective:**

To create and evaluate figures (T-score Maps) to facilitate the interpretation of scores on Patient-Reported Outcome Measurement Information System (PROMIS) measures.

**Methods:**

For 21 PROMIS® short forms, item-level information was used to predict the most probable responses to items for the range of possible scores on each short form. Predicted responses were then “mapped” graphically along the range of possible scores. In a previously conducted longitudinal study, 1594 adult participants with chronic conditions (e.g., multiple sclerosis) responded to four items each of a subset of these PROMIS short forms. Participants’ responses to these items were compared to those predicted by the T-score Maps. Difference scores were calculated between observed and predicted scores, and Spearman correlations were calculated.

**Results:**

We constructed T-score Maps for 21 PROMIS short forms for adults and pediatric self- and parent-proxy report. For the clinical population, participants’ actual responses were strongly correlated with their predicted responses (*r* = 0.762 to 0.950). The majority of predicted responses exactly matched observed responses (range 69.5% to 85.3%).

**Conclusion:**

Results support the validity of the predicted responses used to construct T-score Maps. T-score Maps are ready to be tested as interpretation aids in a variety of applications.

## Introduction

Patient-reported outcome (PRO) measures are increasingly integrated into routine clinical practice to inform clinical decision making [1–3], monitor or screen for symptoms [4, 5], or meet treatment guidelines [6]. In order to base treatment decisions on the PRO scores, providers must be able to accurately interpret their resultant scores. Although guidance on score interpretation was identified by experts as a required component of implementation of PROs in clinical practice [7], a recent systematic review found that only 39% of oncology implementations included it [8]. Approaches to facilitate score interpretation have included identification of important severity thresholds [9–12] and construction of population-based norms reference data [13, 14].

Attributes of the Patient-Reported Outcome Measurement Information System® (PROMIS®) item banks offer potential to create new PRO score interpretation tools. First, in addition to being psychometrically sound [15], PROMIS item banks were developed to reflect how patients conceptualize important symptoms and functions as they apply in one’s day-to-day life. In developing these measures, investigators used mixed methods with substantial patient input [16]. This included identification of important components of a symptom or function to be assessed, as well as reliable and accurate interpretation of the meaning of items across patients [17, 18]. Second, PROMIS measures were constructed with item response theory (IRT) [15, 19]. In IRT, the most likely response to an item can be identified for each score. For example, patients with very poor function are most likely to respond “unable to do” for an item such as, “Are you able to run a short distance such as to catch a bus?” whereas patients with exceptional function are most likely to respond “without any difficulty.” For each item in an IRT-calibrated item bank, a *most likely* response can be identified for each level of the domain measured. This attribute of IRT-calibrated item banks has been used to construct vignettes comprised of subsets of items and responses reflecting different levels of severity [9]. Patients and clinicians have been successful in rank ordering these vignettes, supporting their validity as a tool to convey severity [10–12].

We used IRT-predicted responses for PROMIS item banks to construct figures (“T-score Maps”) that display the most likely responses for a subset of items. This translates numeric scores into language used by patients to describe their degree of severity or impairment in a given symptom or function. Then, we compared the IRT-predicted responses with actual responses in a de-identified archival clinical dataset. We hypothesized that IRT-predicted responses would correlate strongly with patients’ responses (*r* > 0.70) and that the majority of actual responses would be the same as those predicted. We explore potential applications of these figures to facilitate PRO measure score interpretation.

## Methods

### Development of T-score maps

PROMIS measures generate T-scores. T-scores are standard scores with a mean of 50 and standard deviation of 10 in a reference population (usually U.S. general population). T-score Maps were constructed for 21 PROMIS short forms that comprise the PROMIS-57 Profile v2.1, PROMIS Pediatric− 49 Profile v2.0, and PROMIS Parent Proxy-49 Profile v2.0 [20]. The profiles reflect multiple domains of health relevant across the general population and people with chronic conditions, and include highly informative items across mild to severe levels of symptoms and dysfunction. Domains include anxiety, depression, fatigue, physical function, pain interference, sleep disturbance, and social function. Longer short forms (7–10 items) were used in order to represent varied content, allow greater measurement specificity, and be printable on a single page. PROMIS items consist of a statement (e.g., “I feel fatigued”) with five response options (e.g., 1 = not at all, 2 = a little bit, 3 = somewhat, 4 = quite a bit, 5 = very much).

All PROMIS measures were previously calibrated using unidimensional IRT models for each domain [15, 19]. We used the item parameters derived in these calibrations to identify the most probable responses based on the item characteristic curves (ICCs) for each item. ICCs are probability curves that display the probabilities of each response as a function of respondents’ scores on the domain being measured; they are mathematically generated from the IRT model. In ICC plots, probability is plotted on the *y*-axis and scores are plotted on the *x*-axis. For any score on *x*, the response curve with the highest value of *y* is the most probable response. We wrote computer code to identify these most probable responses by score. The code was written using the R program language [21] and is available from the authors. Note that although a response may be the most probable at a given level of severity, this does not necessarily mean that it has a very high probability. A person with a T-score of 60 on PROMIS Anxiety, for example, would have the following response probabilities (*p*) for the item, “My worries overwhelmed me”: never, *p =* 0.089; rarely, *p =* 0.442; sometimes, *p =* 0.415; often, *p =* 0.052; and always, *p =* 0.002. The most likely response is “rarely” but there is an almost equal probability of answering “sometimes”. For a T-score of 61, the response of “sometimes” is the most likely response (never, *p =* 0.063; rarely, *p =* 0.376; sometimes, *p =* 0.484; often, *p =* 0.073; and always, *p =* 0.003). Thus, the most probable response changes from “rarely” to “sometimes” between the T-scores of 60 and 61.

Once the most likely responses at each level of symptom severity or function were obtained for items in the 21 short forms, the results were “mapped” onto the PROMIS T-score continuum in a figure. Specifically, a band for each response option was constructed to indicate the range of scores for which it was the most likely response.

### Comparison of predicted and observed responses

#### Data

Scores predicted by ICCs were compared with observed responses in a de-identified archival clinical dataset. Data came from a survey of adults aging with muscular dystrophy, multiple sclerosis, post-polio syndrome, or spinal cord injury [22]. Individuals living with one of these chronic conditions completed a mailed self-report symptom survey every year for 7 years. Cross-sectional data from year 4 (collected 2012–2013) were used for this secondary analysis because they included the largest sample size for the domains of interest. The dataset included PROMIS v1.0 Fatigue, Anxiety, Depression, and Pain Interference 4a Short Forms (all of which comprise 4 items each). All items in 4a short forms are also included in the short forms displayed in the T-score Map. Of the 1814 surveys mailed, 1594 individuals (88%) completed it. Participants received $25 for completing the survey. All research participants provided informed consent and all study procedures were approved by the University of Washington Human Subjects Division.

#### Analyses

We conducted descriptive analyses to evaluate the degree to which predicted responses matched responses observed in the clinical data. For every participant in the clinical study, we calculated PROMIS T-scores for Fatigue, Anxiety, Depression, and Pain Interference based on their responses to the four administered items of each measure. These T-scores were then located on the appropriate T-score Map. We identified the predicted item response for each item associated with the calculated T-score. We then obtained “difference scores” by subtracting the number associated with their predicted response (1 to 5) from the number associated with their observed response (1 to 5). For example, an individual with a PROMIS Anxiety Score of 60 is predicted to respond “rarely” to, “My worries overwhelmed me.” A response of “rarely” has a numerical value of 2. A respondent who answered “sometimes” (response value of 3), would have a difference score for this item of + 1. Respondents with a T-score of 60 on Anxiety who answered “never” (response value of 1), would have a difference score of − 1. In addition, we calculated the Spearman Correlation Coefficient between predicted and observed responses for each of the 16 items targeted in the study.

## Results

### T-score maps

**Fig. 1:**
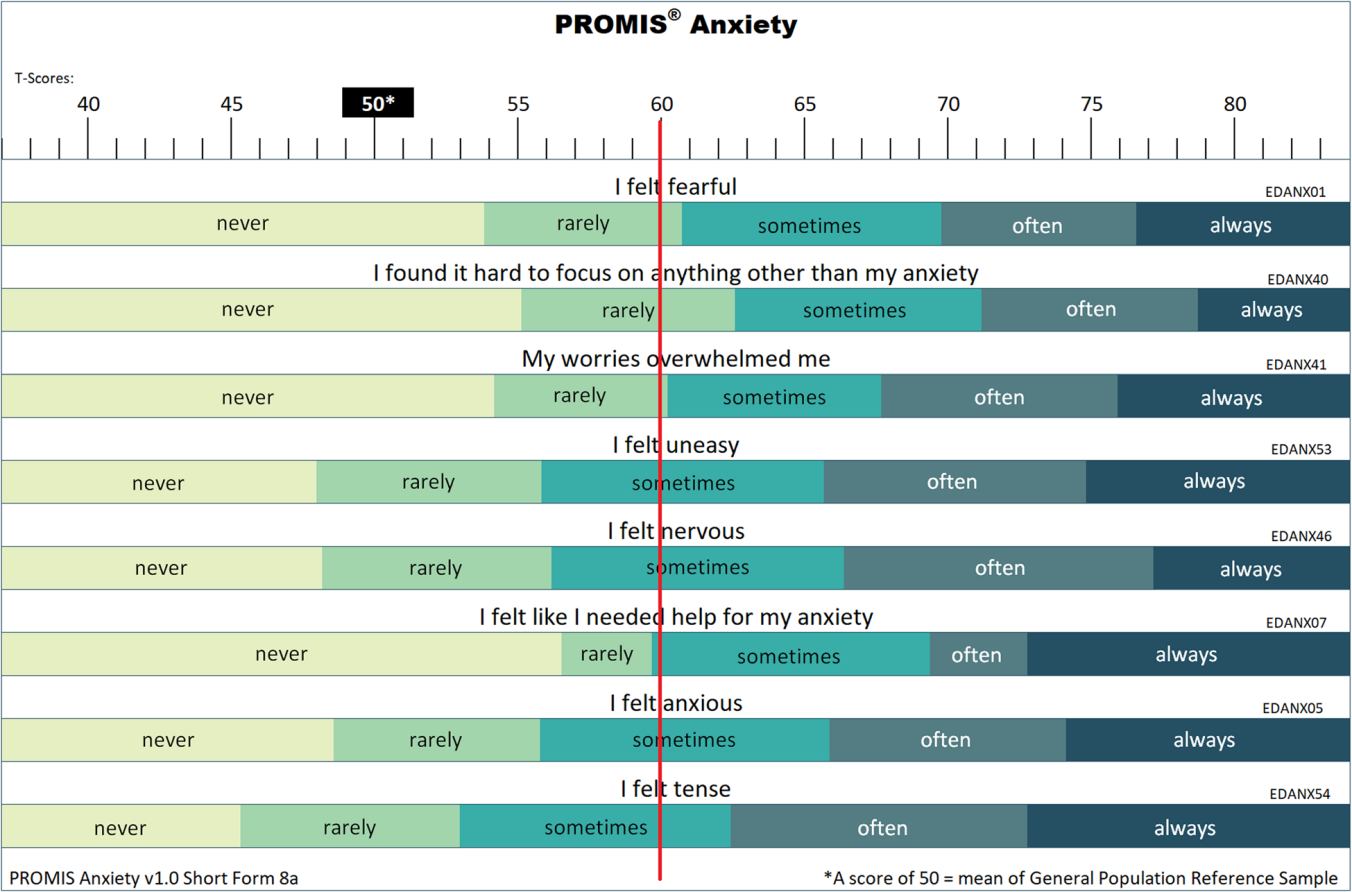
T-score Map for PROMIS Anxiety Short Form 8a with reference line for T-score of 60

We constructed 21 T-score Maps for adult, pediatric, and parent-proxy PROMIS short forms (see Fig. 1). For a given short form, each item was displayed underneath a ruler showing the PROMIS T-score metric. The ranges in which each response category was the most likely response were displayed as shaded bands. As the Fig. 1 Map shows, at T = 60, the most likely response to the item “My worries overwhelmed me” is “rarely;” the most likely response to the item “I felt uneasy” is “sometimes.” All T-score Maps are available at http://www.healthmeasures.net/score-and-interpret/interpret-scores/promis/t-score-maps.

### Sample characteristics

The mean age of the clinical sample was 59.3 years (SD = 13.0), with a mean time since diagnosis of 29.0 years (SD = 21.6). Participants were primarily female (63.8%), non-Hispanic white (91.2%), and had received a college degree or greater (56.7%; Table 1).

**Table 1:** Participant Characteristics

Participant Characteristics (*n* = 1594)	
	Mean [SD]
Participant age (years)	59.3 [13.0]
Years since diagnosis	29.0 [21.6]
PROMIS v1.0 Short Form 4a T-scores	
Fatigue	55.4 [10.4]
Anxiety	51.7 [8.9]
Depression	50.1 [8.8]
Pain Interference	55.0 [9.7]
	n (%)
Diagnosis	
Multiple sclerosis	509 (31.9)
Muscular dystrophy	282 (17.7)
Post-polio syndrome	389 (24.4)
Spinal cord injury	414 (26.0)
Gender	
Female	1017 (63.8)
Male	576 (36.1)
Did not respond	1 (0.1)
Race/ethnicity	
Non-Hispanic White	1454 (91.2)
Other	128 (8.0)
Did not respond	12 (0.8)
Education	
Some high school or less	23 (1.4)
High school grad/GED	189 (11.9)
Some college/vocational or technical degree	476 (29.9)
College degree	499 (31.3)
Graduate or professional degree	405 (25.4)
Did not respond	2 (0.1)

### Comparison of predicted and observed responses

The majority of predicted responses matched the observed responses for each of the 16 items and were consistent across the 4 domains: Fatigue (70.8% to 81.3%), Anxiety (69.5% to 82.0%), Depression (70.5% to 84.9%), and Pain Interference (78.2% to 85.3%). In cases where participants did not select the predicted response, they usually selected the adjacent response reflecting more severity (6.0% to 20.8%) or the adjacent response reflecting less severity (2.5% to 17.1%). These findings were consistent across domains. The IRT-predicted responses displayed in the T-score Maps were strongly correlated with participants’ actual responses to PROMIS short form items (*r* = 0.762 to 0.950, see Table 2). A higher bar to consider is the number of participants whose predicted responses perfectly matched their observed responses across all items of a short form. This level of congruence occurred about half the time with 51.7%, 42.6%, 47.3%, and 55.2% of Fatigue, Anxiety, Depression, and Pain Interference responses matching perfectly across all items of a scale.

**Table 2:** Differences scores (observed - predicted response category) and Spearman correlations between observed and predicted responses

Differences Scores (Observed Response Category - Predicted Response Category) for 16 PROMIS Items and Spearman Correlations between Observed and Predicted Responses									
PROMIS Items	Response Category Differences	Item Content and Correlations							
Fatigue	Observed Response Category - Predicted Response Category	I feel fatigued *r* = .906		I have trouble starting things because I am tired *r* = .874		How fatigued were you on average? *r* = .931		How run-down did you feel on average? *r* = .921	
	Difference	N	Percent	N	Percent	N	Percent	N	Percent
	-4	0	0.0	2	0.1	0	0.0	0	0.0
	−3	0	0.0	5	0.3	0	0.0	1	0.1
	−2	4	0.3	51	3.2	0	0.0	4	0.3
	−1	164	10.3	238	15.0	62	3.9	113	7.1
	**0**	**1174**	**74.0**	**1123**	**70.8**	**1272**	**80.1**	**1290**	**81.3**
	1	240	15.1	163	10.3	251	15.8	175	11.0
	2	5	0.3	5	0.3	3	0.2	4	0.3
	3	0	0.0	0	0.0	0	0.0	0	0.0
	4	0	0.0	0	0.0	0	0.0	0	0.0
	Total N	1587		1587		1588		1587	
Anxiety		I felt fearful *r* = 0.762		I found it hard to focus on anything other than my anxiety *r* = 0.849		My worries overwhelmed me *r* = 0.814		I felt uneasy *r* = 0.848	
	Difference	N	Percent	N	Percent	N	Percent	N	Percent
	−4	0	0.0	0	0.0	0	0.0	0	0.0
	−3	1	0.1	0	0.0	0	0.0	0	0.0
	−2	7	0.4	1	0.1	2	0.1	7	0.4
	−1	132	8.3	53	3.3	95	6.0	271	17.1
	**0**	**1103**	**69.5**	**1302**	**82.0**	**1238**	**78.0**	**1181**	**74.4**
	1	330	20.8	209	13.2	232	14.6	123	7.7
	2	15	0.9	22	1.4	20	1.3	5	0.3
	3	0	0.0	1	0.1	0	0.0	1	0.1
	4	0	0.0	0	0.0	1	0.1	0	0.0
	Total N	1588		1588		1588		1588	
Depression		I felt worthless *r* = 0.847		I felt helpless *r* = 0.798		I felt depressed *r* = 0.846		I felt hopeless *r* = 0.826	
	Difference	N	Percent	N	Percent	N	Percent	N	Percent
	−4	0	0.0	0	0.0	0	0.0	0	0.0
	−3	0	0.0	0	0.0	0	0.0	0	0.0
	−2	12	0.8	5	0.3	4	0.3	1	0.1
	−1	130	8.2	66	4.2	154	9.7	40	2.5
	**0**	**1349**	**84.9**	**1212**	**76.3**	**1120**	**70.5**	**1323**	**83.3**
	1	96	6.0	256	16.1	302	19.0	211	13.3
	2	2	0.1	47	3.0	8	0.5	13	0.8
	3	0	0.0	2	0.1	0	0.0	0	0.0
	4	0	0.0	0	0.0	0	0.0	0	0.0
	Total N	1589		1588		1588		1588	
Pain Interference		How much did pain interfere with your day to day activities? *r* = 0.922		How much did pain interfere with work around the home? *r* = 0.950		How much did pain interfere with your ability to participate in social activities? *r* = 0.916		How much did pain interfere with your household chores? *r* = 0.920	
	Difference	N	Percent	N	Percent	N	Percent	N	Percent
	−4	0	0.0	0	0.0	0	0.0	0	0.0
	−3	0	0.0	0	0.0	0	0.0	0	0.0
	−2	0	0.0	2	0.1	6	0.4	7	0.4
	−1	114	7.2	104	6.6	136	8.7	167	10.7
	**0**	**1253**	**79.5**	**1334**	**85.3**	**1230**	**78.2**	**1222**	**78.2**
	1	200	12.7	121	7.7	185	11.8	151	9.7
	2	9	0.6	3	0.2	13	0.8	14	0.9
	3	1	0.1	0	0.0	2	0.1	1	0.1
	4	0	0.0	0	0.0	0	0.0	0	0.0
	Total N	1577		1564		1572		1562	

## Discussion

PROMIS T-score Maps were constructed for 21 short forms. Each Map displays the most likely responses for possible measure scores. In a follow-up study, predicted responses for a subset of items were compared to responses observed for these items in a clinical dataset and were found to be strongly correlated. This supports the validity of the predicted responses.

Because T-score Maps transform a numeric value to a series of statements about the real-world experience of a symptom or function, they have multiple potential applications. First, they may aid in conveying the meaning of a mean or range of outcomes for various treatments. For example, a clinical trial may identify mean scores for control and intervention groups (e.g., T = 61 versus T = 53). Using Anxiety as an example, with a T-score Map this difference can be conveyed as a “My worries *sometimes* overwhelmed me” to “My worries *never* overwhelmed me.” A clinician and patient can use this information to better understand the expected outcome of a given intervention and inform treatment decisions. A second potential application is to use a T-score Map to set a threshold (e.g., for inclusion in a study, for clinical action). For example, in oncology, collecting PROs for emotional distress is part of standard care. Guidelines state that patients with moderate or severe distress should be provided appropriate referrals for care [23]. T-score Maps for depression and anxiety short forms could be used by mental health experts to aid in identifying thresholds an organization should utilize for referrals. Third, T-score Maps could be utilized as a tool for setting goals for care. For example, a physical therapist may ask patients to identify what level of function the patient hopes to achieve by the end of treatment on a T-score Map. Short form items may be particularly helpful in achieving consensus on treatment expectations because of their ability to convey a range of intensity (e.g., without any difficulty, with a little difficulty, with some difficulty, with much difficulty, unable to do) through their response options. Finally, using T-score Maps to compare two scores could be a helpful tool in creating new methods for identifying what amount of change is meaningful to patients.

This study has three notable limitations. First, the de-identified archival clinical dataset only included four domains (fatigue, anxiety, depression, pain interference) that overlapped with the T-score Map domains. All were adult measures. Although the concordance between IRT-predicted and actual responses was consistent across domains, the extent to which our findings can be generalized to other adult domains or pediatric and parent proxy respondents is untested. Second, the T-score Maps were constructed using primarily 8-item short forms whereas the de-identified archival clinical dataset included 4-item short forms. Although all 4 items were included in the longer short form and the patterns of predicted and actual responses were consistent across items, the extent to which other items from an item bank would produce similar results is untested. Finally, all observed responses were provided by individuals with chronic conditions. Additional comparisons with other samples, particularly those with more emotional health concerns, would clarify the generalizability of our results.

In conclusion, the need for aids in interpreting the meaning of PRO scores is significant. T-score Maps are ready to be tested as interpretation aids in a variety of applications. T-score Maps need not be limited to 4 items and, in fact, those developed for HealthMeasures.net include 7–10 items. T-score Maps that showed predicted responses for all items would be unwieldly because of the number of items that comprise item banks. An interesting line of future study would be to identify items of most relevance to particular patient populations and target these in developing T-score Maps.

## Data Availability

The dataset used in this study is available as a supplemental file. All PROMIS T-score Maps are available at http://www.healthmeasures.net/score-and-interpret/interpret-scores/promis/t-score-maps. R code used to generate response probabilities is available from the authors.

## Abbreviations

IRT: Item response theory
PRO: Patient-reported outcome
PROMIS: Patient-reported Outcomes Measurement Information System
